# Binge Eating Behavior and Weight Loss Maintenance over a 2-Year Period

**DOI:** 10.1155/2014/249315

**Published:** 2014-05-08

**Authors:** Carly R. Pacanowski, Meghan M. Senso, Kristin Oriogun, A. Lauren Crain, Nancy E. Sherwood

**Affiliations:** ^1^Division of Epidemiology & Community Health, School of Public Health, University of Minnesota, 1300 S 2nd Street, Suite 300, Minneapolis, MN 55454, USA; ^2^HealthPartners Institute for Education and Research, 8170 33rd Avenue South, Bloomington, MN 55425, USA

## Abstract

*Objective*. To investigate the relationship between binge eating behavior and weight loss maintenance over a two-year period in adults. *Design*. Secondary data analysis using the Keep It Off study, a randomized trial evaluating an intervention to promote weight loss maintenance. *Participants*. 419 men and women (ages: 20 to 70 y; BMI: 20–44 kg/m^2^) who had intentionally lost ≥10% of their weight during the previous year. *Measurements*. Body weight was measured and binge eating behavior over the past 6 months was reported at baseline, 12 months and 24 months. Height was measured at baseline. *Results*. Prevalence of binge eating at baseline was 19.4% (*n* = 76). Prevalence of binge eating at any time point was 30.1% (*n* = 126). Although rate of weight regain did not differ significantly between those who did or did not report binge eating at baseline, binge eating behavior across the study period (additive value of presence or absence at each time point) was significantly associated with different rates of weight regain. *Conclusion*. Tailoring weight loss maintenance interventions to address binge eating behavior is warranted given the prevalence and the different rates of weight regain experienced by those reporting this behavior.

## 1. Introduction 

In the United States, 34% of adults aged 20 and over are considered overweight, 34% obese, and 6% extremely obese [1]. It is estimated that approximately two-thirds of overweight and obese individuals are currently trying to lose weight [2, 3]. Despite the fact that losing weight and maintaining weight loss can be difficult, research shows that 17–20% of overweight or obese individuals are able to lose weight and maintain their weight loss for at least 1 year [4, 5]. A sustained reduction of 10% of initial body weight is generally considered a success for clinical and research purposes, as this amount, even for very obese persons, can result in numerous health and economic benefits, such as an increase in life expectancy and a reduction in comorbidities such as diabetes, hypertension, and coronary heart disease, along with associated medical costs [5, 6]. Thus, obtaining better understanding of factors that influence successful weight loss maintenance (WLM) is important and could lead to the development of more effective intervention strategies.

The National Weight Control Registry (NWCR) has provided important information regarding correlates of successful weight loss maintenance (defined as having lost at least 13.6 kg (30 pounds) and having kept it off for a minimum of one year). The NWCR is a group of over 3,000 individuals who have lost an average of 30 kg and maintained this loss for an average duration of 5.5 years [5]. Data from the NWCR indicates that successful weight loss maintainers have high physical activity levels, eat a diet low in fat and high in carbohydrate, and regularly self-monitor their weight, while higher levels of depression, disinhibition, and binge eating increase the risk of weight regain [5], suggesting that binge eating may be a behavior important to successful WLM.

Although considerable research examining the relationship between binge eating and weight loss has been conducted [7–11], there is a paucity of research on the relationship between binge eating and prolonged WLM. Of interest, rates of binge eating in NWCR participants are comparable to those observed in community samples, with 8% of members reporting 4 or more binges per month [12]. Beyond this observational study, empirical evidence on the relationship between binge eating and WLM is scarce. The weight loss literature on this topic is mixed. Most studies have not found binge eating to be a strong predictor of weight loss success [7–11]. However, one study found that binge eaters who abstained from binging earlier in the program had significantly greater weight loss than those who continued binge eating [13] and several other studies have found that individuals who engage in binge eating regain weight more rapidly after treatment compared to nonbingers [9–11]. It has also been documented that participants who binge eat are more likely to drop out of treatment programs [7, 9–11], which may mask potential relationships that exist between binge eating and weight loss outcomes.

Although there is limited support for binge eating as a predictor of weight loss, binge eating behavior may be negatively associated with WLM, based on evidence from the studies cited previously, along with the evidence that successful weight maintainers report low rates of binge eating [12] and individuals who binge eat have been shown to gain more weight over time [14].

The Diagnostic and Statistical Manual of Mental Disorders 5th edition (DSM-5) now includes binge eating disorder as a psychiatric illness in recognition of the psychological distress and impaired functioning that is associated with this behavior. A binge eating episode is defined as consumption of a subjectively large amount of food and sense of loss of control over eating during this episode [15]. To meet the DSM-5's criteria for BED, an individual must engage in a binge eating episode at least 1 time per week for 3 months [15]. Furthermore, the DSM-5 denotes classifications for severity of binge eating: if 1–3 binge episodes occur weekly, this is classified as mild binge eating disorder. BED of moderate severity occurs at a frequency of 4–7 episodes per week, severe at a frequency of 8–13 episodes per week, and extreme if 14 or more episodes occur weekly [15]. The prevalence of BED in individuals seeking treatment for obesity has been reported to be as high as 25%, compared to estimates of 2–5% from community samples [12].

Although binge eating behavior has been studied extensively, controversy exists about how to best operationalize the disorder in a research setting [16]. Many studies have measured binge eating at a sole time point (e.g., [7, 14]) while few studies have examined binge eating over time [17, 18]. This is important because it is possible that binge eating behavior fluctuates over time and a single time point may not capture all experiencing symptoms. Though data has been used to assert that binge eating behaviors remain stable [18], other research shows that certain disordered eating behaviors, like binge eating, fluctuate over time [17]. Though Hawkins and Clement [18] reported temporal stability of binge eating (test retest = 0.88), their sample consisted of undergraduates, most of whom were of normal weight, and the binge eating scale utilized had only moderate internal consistency (0.68). Moreover, the period of assessment for stability was five weeks and it is possible that cycles of binge eating exceed five weeks. Consequently, it may be important to measure binge eating at multiple time points in order to more accurately assess the relationship between binge eating and weight change, particularly among adults who are trying to maintain a clinically meaningful weight loss.

An important and potentially confounding factor to consider when studying binge eating is depression. Though depression and binge eating are not interchangeable, it has been suggested that there is a relationship between the two [19]. Empirical data has supported this relationship, especially in women [8]. A number of previous studies have found depression to be associated with both binge eating [9, 20–22] as well as with weight status [23, 24]. More recently, negative affective states have been shown to precede binge eating in overweight and obese adults [25]. Thus, measuring and attempting to prevent depression score from confounding the relationship between binge eating behavior and weight loss maintenance may be advisable.

The current study addresses a gap in the literature by examining the relationship between binge eating behavior and WLM among adults who recently lost at least 10% of their body weight and enrolled in a study to test the effect of an intervention on WLM success. Because binge eating may fluctuate over time and this may influence WLM, binge eating behavior was assessed at multiple time points. Binge eating behavior was measured at baseline, 12 months, and 24 months, allowing for both cross-sectional and longitudinal approaches to categorizing this behavior. We hypothesize that (H1) individuals who report binge eating behaviors at baseline will have a greater rate of weight regain over the 24-month trial compared to individuals who do not report binge eating at baseline. Additionally, accounting for binge eating status at multiple time points, taking a longitudinal approach to this construct, will be informative beyond the use of baseline binge eating status alone. Specifically, we hypothesize that (H2) participants who report binge eating at multiple time points will have a greater rate of weight regain over the 24-month trial relative to participants who report no binge eating or less consistent binge eating (reporting binge eating at one out of 3 time points). Because of its potential role in the relationship between binge eating and weight regain, depression was included in analyses.

## 2. Materials and Methods 

### 2.1. Participants

Keep It Off (KIO) participants are members of the Minnesota-based HealthPartners managed-care organization who intentionally lost at least 10% of their body weight during the previous year. Briefly, a participant's progression through the study was as follows: investigators recruited participants who had lost at least 10% of their body weight in the past year, interested individuals were telephone-screened for eligibility (*n* = 875), and those who met inclusion criteria were enrolled and randomized to one of two weight loss maintenance groups (*n* = 419) and then received either the self-directed or guided weight loss maintenance intervention. Participants in the self-directed intervention received less education about weight loss maintenance (two 20-minute phone calls and a self-monitoring logbook to keep track of progress) compared to the guided intervention (twenty-four 20-minute phone calls plus followups along with the self-monitoring logbook which they were required to submit at regular intervals to intervention staff) [26].

During eligibility screening, participants were asked to describe how (e.g., weight loss program or decreasing caloric intake on their own) and why they purposefully lost weight. Inclusion criteria for participation in the study were as follows: 19 to 70 years old, BMI > 20.5 kg/m^2^, and the capacity to communicate with research staff by telephone. Participants could not have a history of anorexia nervosa, previous bariatric surgery, recent diagnosis of a non-skin cancer or congestive heart failure, and/or current participation in another phone-based weight loss program or in another weight-management study. The KIO intervention and primary outcomes are described in detail elsewhere [26, 27].

### 2.2. Measures

#### 2.2.1. Weight and Height

Weight and height were measured at baseline, 12 months, and 24 months during in-person visits with subjects wearing light clothes and without shoes (Seca 770 Medical Scale; Seca 214 Portable Height Rod). Body Mass Index (BMI) was calculated from this measure in addition to an in-person height measurement.

#### 2.2.2. Binge Eating Behavior

Binge eating behavior was self-reported via survey at baseline, 12 months, and 24 months during in-person visits and defined using three items from the Eating Disorder Diagnostic Scale (EDDS): (1) eating what other people would regard as an unusually large amount of food “Have there been times when you felt that you have eaten what other people would regard as an unusually large amount of food (e.g., a quart of ice cream) given the circumstances?” (Response options = yes/no); (2) a perceived loss of control during these episodes “During the times when you ate an unusually large amount of food, did you experience a loss of control (feel you could not stop eating or control what or how much you were eating)?” (Response options = yes/no); and (3) the frequency of binge episodes “How many TIMES per week on average over the past 6 months have you eaten an unusually large amount of food and experienced a loss of control?” (Response options = 0, 1, 2,…13, 14+) [28]. The EDDS has good psychometric properties: internal consistency: alpha = 0.89; test-retest reliability: *r* = 0.87 [28, 29].

#### 2.2.3. Categorizing Binge Eating Behavior

At baseline and at 12 and 24 months, each participant was categorized as a binge eater if they reported eating a large amount of food and feeling a loss of control over eating at least once per week on average over the past 6 months. In cases of missing data, if it could not be verified that the participant self-reported this behavior at least once a week, in accordance with DSM-5 criteria, they were labeled as missing or not experiencing binge eating depending upon the other information available. For example, some participants may have reported eating a large amount and skipped the question about experiencing a loss of control but reported a frequency of an average of >1 times per week; these participants were classified as engaging in binge eating because they reported a frequency specified by the DSM-5, and the final question clearly states episodes where both a large amount and a loss of control were experienced.

Binge eating behavior was categorized as being present or absent at each time point (baseline, 12 months, and 24 months). For H1, we were interested in how binge eating behavior at baseline was associated with rate of weight regain over two years. For H2, we were interested in how the consistency of binge eating behavior at multiple time points (e.g., an individual may not have reported binge eating at baseline but may have reported binge eating at 12 months and/or 24 months) was associated with rate of weight regain over two years.

#### 2.2.4. Binge Eating Behavior at Baseline

H1 is concerned with whether participants met the criteria for binge eating at baseline. All analyses pertaining to this hypothesis used the aforementioned definition of binge eating behavior and applied it to baseline only.

#### 2.2.5. Binge Eating Behavior over Time

*Any Binge Eating*. Assessment of binge based on assessments at multiple time points may provide different estimates of the occurrence of this behavior relative to a single point in time, such as baseline. For this reason, a variable was created to assess whether participants met criteria for binge eating behavior at any data collection time point throughout the two-year study. This is a dichotomous variable—if a participant met the aforementioned criteria for binge eating at baseline or 12 months or 24 months, they would be classified as having binge eating behavior. As shown in Table 2, nearly a third of the sample self-reported binge eating behavior during at least one time point during the two years of the study. 

*Recurrent Binge Eating*. To address H2, a cumulative binge score assessing recurrent binge eating was created; this score is the sum of self-reported binge eating behavior at each time point with a minimum of 0 indicating no binge eating at any time point and a maximum of 3 indicating a participant self-reporting binge eating at all three time points (baseline, 12 months, and 24 months). A three-level categorical variable was then created for individuals reporting no binge eating at any time points, binge eating behaviors at one time point, or binge eating behaviors at 2 or more time points. Since a score of 2 meant reporting binge eating more often than not (2 out of 3 possible time points), we classified them as consistent binge eaters and those with a score of 1 as inconsistent binge eaters. This variable was treated as categorical in the modeling.

#### 2.2.6. Severity of Binge Eating Behavior

Based on the new DSM-5 definitions for binge eating severity, severity of binge eating was categorized for each of the time points at which a person was classified as a binge eater. As discussed, if 1–3 binge episodes occur weekly, this is classified as mild binge eating disorder. BED of moderate severity occurs at a frequency of 4–7 episodes per week, severe at a frequency of 8–13 episodes per week, and extreme if 14 or more episodes occur weekly [15].

#### 2.2.7. Depression

The 11-item short form of the Center for Epidemiological Studies Depression (CES-D) symptoms scale was used to measure depression via in-person survey at baseline, 12 months, and 24 months. This version of the CES-D has been shown to have high internal consistency and correlates strongly with the original 20-item CES-D scale that is broadly accepted by researchers and clinicians [30]. The 11-item scale has a maximum possible score of 33. A participant's score on the CES-D is the sum of responses to 11 items (e.g., “I felt sad”; rated on a scale of “rarely or none,” “some,” “moderate,” and “all”) regarding the amount of time at which they felt they agreed with the statement in the past week, with two items reverse-coded. A score of 9 or higher on the 11-item scale corresponds with a score of 16 or higher on the 20-item scale and represents clinically significant symptoms of depression [31].

Additional demographic variables used in the analyses included gender and age.

#### 2.2.8. Statistical Analysis

Measures of central tendency and dispersion were calculated for demographic variables, depression, binge eating, and body weight for the study sample and then stratified by baseline and by consistency of binge eating behavior. *t*-tests and analysis of variance compared means between groups for continuous variables, and chi-square tests compared proportions between groups for categorical variables.

Hypotheses 1 and 2 were tested by estimating mixed models to predict three weight measures per person from two key predictors and their interaction: baseline binge eating status/consistency of binge eating status and the time at which weight was measured (baseline, 12 m, and 24 m). Main effects for treatment group assignment, depression, age at baseline, and gender were included as covariates, as well as the treatment by time interaction to control for intervention efficacy. Interactions between binge eating and depression and between treatment group, time, and binge status were included in preliminary models to ensure that the effects of primary interest were not modified by other modeled covariates. Neither interaction was significant and thus not retained in the final models. The nonsignificant interaction between binge eating and depression indicated that participants that were more depressed did not show a significantly different relationship between binge eating and weight over the two years compared to participants who were less depressed. Study participant was the unit of analysis in these models, with repeated weight observations nested within participants. Two random effects, the weight intercept and the time slope, were estimated for each participant with the remaining parameters treated as fixed.

The prediction that binge eaters would regain weight at a faster rate over the two-year period would be most strongly supported by a significant interaction between binge status and time. The key difference between the H1 and H2 models was that binge eating behavior had two values (i.e., not a binge eater at baseline and binge eater at baseline) in the H1 model but three (i.e., not a binge eater at any time, inconsistent binge eater, and consistent binge eater) in the H2 model.

All statistical analyses were completed using Statistical Analysis System (SAS) version 9.3 and the Statistical Package for the Social Sciences (SPSS) version 20 [32, 33].

## 3. Results

### 3.1. Participant Characteristics

Table 1 presents baseline demographic and psychosocial characteristics for the sample. The majority of participants were Caucasian, married, employed, nonsmoking females with an average age of 46.5 years. The average BMI was 28.5 kg/m^2^, classifying most participants as overweight despite recent weight loss of at least 10% of body weight. Average baseline depression scores were 5.5, indicating nonclinically significant depression symptoms.

Prevalence of self-reported binge eating is presented in Table 2 for baseline, 12 months, 24 months, and any of the three time points. Binge eating behavior was self-reported by about one-fifth of the sample at baseline and 12 months; the proportion of the sample reporting binge eating behavior was about one out of six at 24 months. About 30% of the sample met this criterion when considering any time point. As shown in Table 2 the majority (three-quarters or greater) of participants reported binge eating episodes that would be classified as mild, occurring between 1 and 3 times per week. Binge eating behavior was missing for 28 participants at baseline (6.7% of the sample), 76 participants at 12 months (18.1% of the sample), and 80 participants at 24 months (19.1% of the sample).

Table 3 presents demographic and descriptive statistics for the study sample, stratified by binge eating behavior at baseline and by binge eating behavior consistency (no binge, inconsistent binge, or consistent binge). Significant differences observed between those not reporting binge eating behaviors and those reporting binge eating behaviors at baseline included weight, BMI (calculated using weight), baseline depression, and average depression score, all of which were higher in those reporting binge eating behavior. BMI was not significantly different between genders at any time point but was significantly, but weakly positively, associated with both baseline depression and average depression at each time point (*P* < 0.05; *r*-values: 0.109–0.217).

Stratifying by binge consistency shows that approximately 69.9% of participants reported no binge eating behavior, 17.7% reported inconsistent binge eating behavior (reported binge eating at one time point), and 12.4% were consistent binge eaters (reported binge eating at two or three time points). Similar to the comparisons made by baseline binge status, these categorizations are associated with significant differences in baseline BMI and depression scores. Baseline depression score was not significantly different between groups of binge eating consistency in this analysis; however age was such that those reporting no and consistent binging were significantly older than those reporting inconsistent binging.

### 3.2. Binge Eating Behavior and WLM

**Figure 1:**
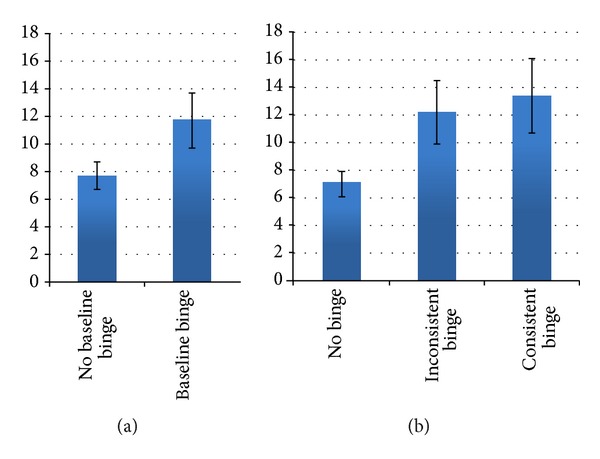
Two-year weight change (lbs) by (a) baseline binge eating status and (b) binge eating category. Error bars represent ±1 standard error.

Figure 1 presents descriptive results of weight change over 24 months for (a) those who reported presence or absence of binge eating at baseline (no baseline binge eating corresponded with an average gain of 7.7 pounds (3.5 kg); baseline binge eating corresponded with an average regain of 11.7 pounds (5.3 kg)) and (b) those who reported no binge eating, inconsistent binge eating, or consistent binge eating (average regains of 7.0 pounds (3.2 kg), 12.2 pounds (5.5 kg), and 13.4 pounds (6.1 kg), resp.), supporting the hypothesis that binge eating is associated with more weight regain.

**Figure 2:**
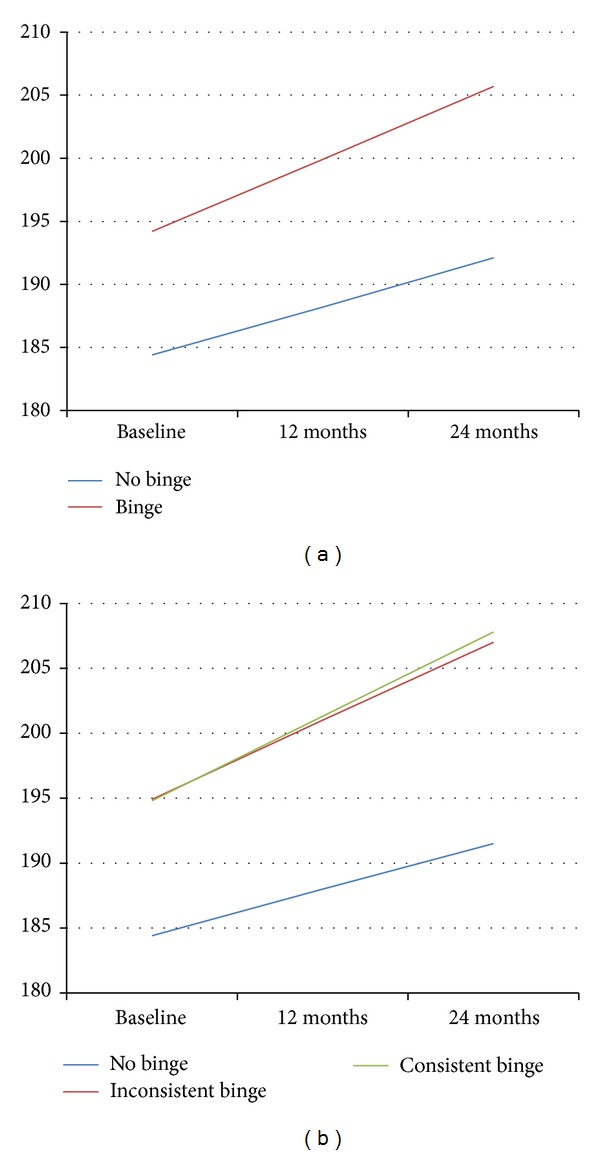
Weight (lbs) by (a) baseline binge eating status and (b) binge eating consistency.

Figure 2 displays the model-estimated weight gain trajectories by (a) baseline binge eating behavior and (b) binge consistency. In both graphs, those that report binge eating are heavier at baseline, corroborating significant differences reported in Table 3. In Figure 2(a), individuals who report no binge eating behavior at baseline gain roughly 3.8 pounds (1.7 kg) per year while individuals who report binge eating behavior at baseline gain about 5.7 pounds (2.6 kg) per year (*P* value for time < 0.001). The difference in rate of regain was in the predicted direction but did not reach conventional levels of statistical significance (*P* < 0.08, pseudo-*R* ^2^ = 0.545).

As for the additional parameters that were estimated, the intervention by time interaction was statistically significant (*P* = 0.011), which is consistent with the primary outcome paper result that participants in the guided versus self-directed intervention arm regained weight at a slower rate over the 24-month followup [26]. Baseline depression and gender were significant main effects (*P* = 0.006 and <0.001, resp.) meaning that more depressed people weighed more than less depressed people and women weighed less than men. Age was not related to weight regain (*P* = 0.08).

Figure 2(b) depicts the weight trajectories for groups categorized by binge consistency. The yearly rate of regain for participants that did not meet criteria for binge eating at any of the three time points was 3.6 pounds (1.6 kg) on average, for those reporting binge eating at one time point was 6.1 pounds (2.8 kg) on average, and for those reporting binge eating at 2 or 3 time points was 6.5 pounds (3.0 kg) on average. In contrast to the first model that only considered binge eating status at baseline, this model reveals that participants who did not report binge eating, reported inconsistent binge eating, and reported consistent binge eating across multiple time points had significantly different weight regain trajectories over the 2 year study period (*P* = 0.013, pseudo-*R* ^2^ = 0.521). The effect appears to be driven by the two binge groups relative to the group reporting no binge eating.

Models were rerun using BMI as an outcome. Weight and BMI trajectories followed similar patterns and significance levels of terms were very close to the reported *P* values. For ease of interpretability, weight was chosen as the outcome.

## 4. Discussion

This is one of the first studies to assess the relationship between binge eating behaviors and weight loss maintenance. Participants in this study had lost at least 10% of their body weight in the past year and were randomized to either a self-directed program or a guided intervention designed to help maintain weight loss. Results show that binge eating behavior is associated with greater weight regain independent of the effect of the WLM intervention.

Numerous individuals reported binge eating at baseline and reported no binge eating at future evaluations and vice versa. In response to this observation, a new variable was created to capture binge eating consistency throughout the study. Data were modeled separately using baseline binge eating behavior and the measure of binge eating consistency to examine how results might differ according to whether only baseline binge eating is considered or a more longitudinal proxy for binge eating behavior is used. Concerning our first hypothesis, when considering only baseline binge eating, rate of weight regain was not statistically different (*P* = 0.077) between groups, though differences in rates were in the hypothesized directions as depicted by Figure 2(a). When using the newly created variable to address binge eating behavior consistency throughout the study, differences in rate of weight change were more pronounced and were statistically significantly different (*P* = 0.013), supporting our second hypothesis. This could be due to increased power to detect an effect or a truly increased rate of weight regain when taking binge behavior at any time point into account. Both baseline and average depression scores were significantly related to weight change over 2 years. The effect of the treatment group on weight regain over time was also substantial, as those randomized to the self-directed group regained significantly more weight by the 24-month followup compared to those in the guided maintenance intervention. However, no significant interaction was found between depression and binge eating, or binge eating, treatment group, and time, as binge eating was equally detrimental for those in both treatment groups. This may be a result of the fact that the intervention focused more broadly on weight loss maintenance strategies and did not specifically target binge eating behaviors.

The present investigation has several limitations. First, because the primary goal of the Keep It Off study was to evaluate the effectiveness of a WLM intervention, data may not have been collected in a manner that would most accurately assess binge eating behavior. Three questions were used from the Eating Disorder Diagnostic Scale along with guidance from newly defined DSM-5 criteria to determine whether an individual self-reported engaging in binge eating behavior. The EDDS is subject to limitations inherent in any self-report measure: retrospective recall bias, memory (especially over a 6-month period), and distortion or inaccurate reporting due to desire to please the experimenter or social desirability. However, in one study self-report has shown to have adequate validity compared with the gold standard interview method for assessing binge eating disorder behaviors [34]. Another limitation includes inability to assess directionality of binge eating and weight change due to impracticably small numbers of participants grouped according to the direction of change in binge status. Trends were in the expected direction, as individuals who reported binging at the end but not at the beginning of a year gained more weight than those reporting binging at the beginning but not at the end of the year. Future research investigating directionality may be designed to better investigate “binge cycles” using clinical populations.

Finally, the sample recruited for this study was not representative, as participants were mostly Caucasian (86.87%) and female (81.62%). Previous research shows that, unlike other eating disorders, BED is distributed fairly equally among women and men. Research also shows that differences exist in the rates and severity of binge eating, as well as in the psychological correlates of binge behaviors by ethnicity. It would be advisable for future research on binge eating to include greater proportions of male subjects and ethnic minorities.

Results from this study demonstrate primarily that individuals reporting engaging in binge eating behaviors regain weight at a faster rate than those who do not report binge eating behaviors. For future studies, assessing binge eating behavior at multiple time points may be helpful in examining the magnitude and change in binge eating behaviors over time. This may capture those individuals who do not meet the full criteria for BED, but who engage in a level of subclinical binge eating behaviors that could affect health outcomes.

Based on the findings presented here, binge eating behaviors affect a substantial percentage of individuals that have lost weight and are trying to prevent weight regain. Binge eating behaviors were shown, in this study, to significantly affect the amount and rate of weight regained over a two-year period. As mentioned, little attention has been paid to the role of binge eating in weight loss maintenance. Work has been done looking at different treatment modalities for individuals experiencing binge eating and suggests that cognitive behavioral therapy (CBT) works to decrease symptomology but does not substantially reduce body weight while standard weight loss therapy is more successful in terms of amount of weight loss during the active treatment phase [35–38]. However, as time progresses after treatment, recurrence of binge eating symptoms may contribute to weight regain, which could be one factor contributing to nonsignificant weight differences between treatment groups at followup [35]. Additionally, interpersonal therapy (IPT) has been studied in treating BED and found to reduce symptoms [39] and performed significantly better than behavioral weight loss [40]. IPT may be a promising treatment modality to include when treating BED. Developing interventions that offer more tailored support is essential in moving forward to promote weight loss maintenance in the appreciable portion of individuals who have lost weight and experience binge eating behavior.

## Figures and Tables

**Table 1:** Baseline demographic and psychological data for entire study population.

Demographics, weight, and psychological characteristics	Mean (SD) or %
Age (y)	46.5 (10.8)
Weight (lbs)	175.9 (35.5)
Gender (% female)	81.6
Treatment group (% guided)	49.9
Body Mass Index (kg/m^2^)	28.5 (4.9)
Ethnicity (% non-Hispanic white)	91.4
Employment status (% employed)	87.1
Depression	5.2 (3.8)
Marital status	
Married	67.9
Divorced, separated, or widowed	17.9
Never married	14.1

**Table 2:** Number and percentage of participants self-reporting binge eating behaviors by evaluation time point and severity.

	Baseline (*n* = 391)	12 months (*n* = 343)	24 months (*n* = 339)	Any time point (*n* = 418)
No binge (%)	315 (80.6)	275 (80.2)	285 (84.1)	292 (69.9)
Binge eating behavior (%)	76 (19.4)	68 (19.8)	54 (15.9)	126 (30.1)

	Baseline (*n* = 76)	12 months (*n* = 68)	24 months (*n* = 54)	

Severity of binge eating behavior *n* (%)				
Mild	57 (75.0)	56 (82.4)	44 (81.5)	
Moderate	16 (21.1)	10 (14.7)	8 (14.8)	
Severe	3 (3.9)	1 (1.5)	1 (1.9)	
Extreme	0 (0.0)	1 (1.5)	1 (1.9)	

**Table 3:** Demographic and psychological characteristics stratified by baseline binge status and overall binge status.

Demographics, weight, and psychological characteristics	No baseline binge mean (SD) or % (*n* = 315)	Baseline binge eating mean (SD) or % (*n* = 76)	*P*	No binge mean (SD) or % (*n* = 292)	Inconsistent binge mean (SD) or % (*n* = 74)	Consistent binge mean (SD) or % (*n* = 52)	*P*
Age (y)	47.0 (10.4)	45.0 (11.7)	0.145	47.4 (10.3)	42.6 (11.4)	47.2 (11.1)	0.002*
Weight (lbs)	173.7 (33.5)	183.2 (39.5)	0.034*	173.3 (33.6)	181.5 (40.0)	183.9 (37.0)	0.048*
Body Mass Index (kg/m^2^)	28.1 (4.5)	29.6 (5.5)	0.028*	27.9 (4.8)	29.6 (5.5)	29.5 (4.9)	0.007*
Gender							
% Female	81.0	82.9	0.697	79.5	87.8	84.6	0.209
Treatment group							
% Self-directed control	49.2	52.6	0.592	47.3	54.1	61.5	0.127
Ethnicity							
% non-Hispanic white	92.8	90.7	0.536	92.3	88.6	92.2	0.598
Employment status							
% Employed	84.8	77.6	0.135	82.9	86.5	73.1	0.136
Marital status							
% Married	66.8	65.3	0.553	67.1	63.2	66.7	0.570
% Previously married	19.8	16.7		20.0	17.6	14.6	
% Never married	13.4	18.1		12.9	19.1	18.8	
Baseline CES-D depression score	4.8 (3.5)	6.1 (4.0)	0.007*	4.9 (3.5)	5.7 (4.8)	5.9 (3.7)	0.077
Average CES-D depression score	5.1 (3.5)	6.2 (3.8)	0.019*	5.0 (3.4)	6.5 (4.5)	6.4 (3.6)	0.001*
Withdrawal status (%)							
Yes	8.3	6.6	0.628	8.6	4.1	5.8	0.371

*P for difference <0.05.
